# Identification and Transcriptome Resource of the Mite *Orthogalumna* cf. *terebrantis* (Acari: Galumnidae) in China

**DOI:** 10.3390/cimb48060619

**Published:** 2026-06-15

**Authors:** Menghui Yang, Xiaochuan Ma, Konglin Zhou, Sheng Lin, Jianming Chen, Zhenyue Lin

**Affiliations:** 1 College of Plant Protection, Fujian Agriculture and Forestry University, Fuzhou 350002, China; 12362003003@fafu.edu.cn (M.Y.);; 2 Fujian Key Laboratory of Conservation and Sustainable Utilization of Marine Biodiversity, College of Geography and Oceanography, Minjiang University, Fuzhou 350108, China; e1597915@u.nus.edu (X.M.); linzkl@163.com (K.Z.); 3 Technology Innovation Center for Monitoring and Restoration Engineering of Ecological Fragile Zone in Southeast China, Ministry of Natural Resources, Fuzhou 350001, China

**Keywords:** *Orthogalumna*, SEM, 18SrDNA, transcriptome

## Abstract

The genus *Orthogalumna* (Oribatida: Galumnidae) has been recognized for its phytophagous associations with aquatic plants, particularly its potential role in the biocontrol of the invasive weed Water hyacinth (*Eichhornia crassipes*). Despite its ecological significance, this genus remains poorly studied in terms of its micromorphological architecture, phylogenetics, and genomic resources. In this study, we report *Orthogalumna* cf. *terebrantis* from China, providing the first comprehensive characterization of an *Orthogalumna* species by integrating morphology, phylogeny, and transcriptomics. This record represents the first documented occurrence of *O.* cf. *terebrantis* in China, pending confirmation by voucher-based morphological comparison and molecular data. This work provides critical microstructural evidence to complement traditional morphological identification and establishes a foundational molecular dataset for future studies on the systematics, comparative genomics, and environmental adaptation of oribatid mites.

## 1. Introduction

Water hyacinth (*Eichhornia crassipes* (Mart.) Solms) is an invasive plant from South America that represents one of the most pernicious and destructive invasive species globally [1]. Its rapid growth harms local biodiversity and water ecology systems [2,3]. The genus *Orthogalumna* (Oribatida: Galumnidae) is notable for its specialized phytophagous ecology and its potential application as a classical biological control agent. Most oribatid mites are terrestrial detritivores or fungivores, inhabiting soil and leaf litter [4,5,6,7]. However, *Orthogalumna* is exceptional in its strict ecological association with aquatic macrophytes. *Orthogalumna terebrantis* further compromises host performance through the feeding galleries produced by females and their larvae, which exacerbate damage to plant structural integrity and photosynthetic capacity [8]. When this mite acts together with other host-specific biological control agents [9], especially the weevils *Neochetina eichhorniae* and *Neochetina bruchi*, it adds to a cumulative stress effect that gradually weakens the competitive dominance of water hyacinth and helps native aquatic vegetation recover, along with ecosystem function. This approach has been used in countries such as Argentina [10], India [11], and South Africa [12]. Even so, its true diversity and the evolutionary mechanisms that support adaptation to different microhabitats are still not fully understood. This research gap is particularly pronounced in the genus *Orthogalumna*, where studies have traditionally depended on morphological analysis. Consequently, the development of genetic resources represents a crucial area requiring immediate attention.

High-throughput sequencing-based transcriptomics is a powerful tool that provides comprehensive gene expression profiles and molecular insights, particularly for non-model organisms without reference genomes [13,14,15]. For instance, studies on *Dermanyssus gallinae* and *Ornithonyssus sylviarum* have focused on the diversification of chemoreceptor gene families, revealing adaptations to host–seeking behavior [16,17]. In parallel, comparative transcriptomics of *Dermatophagoides pteronyssinus*, *Alaskozetes antarcticus*, *D. gallinae*, and *Tetranychus urticae* have provided insights into reproductive strategies across free-living and parasitic lifestyles [18,19,20,21]. Furthermore, transcriptome analyses of *Amblyseius swirskii*, *T. urticae*, *Eotetranychus sexmaculatus*, and the pink tea mites have uncovered genes mediating mite-host interactions and defense responses, contributing to our understanding of plant-herbivore co-evolution [22,23,24,25]. These studies have facilitated the discovery of functional genes, metabolic pathways, and evolutionary relationships.

Here, we report *O.* cf. *terebrantis* in China through an integrative approach that combines detailed morphology identification, molecular phylogenetics, and transcriptomic sequencing. By employing high-resolution scanning electron microscopy (SEM), we provide a detailed elucidation of its intricate morphology, offering clear visualizations of taxonomically significant characters. Phylogenetic reconstruction based on 18S rDNA gene sequences robustly places *O.* cf. *terebrantis* within the *Galumnidae* lineage and clarifies its evolutionary relationships within *Oribatida*. In addition, we generate the first de novo transcriptome resource for *O.* cf. *terebrantis*.

## 2. Materials and Methods

### 2.1. Sample Collection and SEM Analysis

This study was conducted from 2024 to 2025 in a river channel at Minjiang University in southeastern Fujian Province, China (26.0730° N, 119.1621° E). Mite sampling focused on the invasive plant water hyacinth, which densely covered extensive areas of the river. Plants were collected using an extendable pole, transported to the laboratory, and adults and nymphs inhabiting them were subsequently extracted using fine-pointed insect pins.

For SEM analysis, *O.* cf. *terebrantis* specimens were fixed in 2.5% glutaraldehyde at 4 °C overnight in darkness. After fixation, the specimens were washed three times with 0. 1 M phosphate-buffered saline (PBS, pH 7.4) for 10 min each, followed by sequential dehydration through a graded ethanol series of 30%, 50%, 70%, 80%, 85%, 90%, 95%, 100%, and 100% for 10 min at each step. Following critical-point drying with a CO_2_ critical-point dryer (Quorum K850: Quorum Technologies Ltd., Laughton, UK)), the dehydrated specimens were mounted onto aluminum stubs using conductive double-sided adhesive tape and examined under a stereomicroscope. Following a 60 s gold sputter coating in an ion coater (SuPro J20: SuPro Instruments Co., Ltd., Suzhou, China), the samples were viewed under a scanning electron microscope GeminiSEM 360: Carl Zeiss Microscopy GmbH, Oberkochen, Germany to get whole-body pictures and detailed morphological features of the adult mites. Morphological terminology used in this study follows the general concepts outlined by Ermilov and Klimov for the family Galumnidae [5].

### 2.2. Phylogenetic Analysis

#### 2.2.1. DNA Extraction and 18S rDNA PCR Amplification

Genomic DNA was extracted from 50 specimens using the TIANamp Marine Animals DNA Kit according to the manufacturer’s instructions. Primers for 18S rDNA were 5′-GAAACGGCT ACCACATCC-3′(18Sforward) and 5′-TTCGCTTTAGTTCGTCTTGC-3′(18Sreverse). The target genes were amplified in a 25 µL reaction volume. The PCR cycling conditions were as follows: 3 min of denaturation at 94 °C; 35 cycles of 30 s of denaturation at 94 °C; 30 s of annealing at 42–55 °C (depending on the primers); 1 min of extension at 72 °C; 5 min final extension at 72 °C; and holding at 4 °C. The 18S rDNA fragment was amplified at 52 °C using standard PCR protocols. PCR products were separated by electrophoresis on 1.0% agarose gels. Primer synthesis and sequencing of the 18S PCR products were conducted by Sangon Biotech (Shanghai, China). Newly generated 18S rDNA sequences have been deposited in GenBank.

#### 2.2.2. Sequence Alignment and Phylogenetic Analysis

18S rDNA raw forward and reverse sequences were assembled by Sangon Biotech (Shanghai, China). To construct a comprehensive phylogenetic data matrix, the 18S rDNA sequence of *O.* cf. *terebrantis* was aligned with 80 previously published oribatid mite sequences using ClustalW implemented in MEGA v7 [26,27]. All 18S rDNA sequence data were obtained from GenBank. Phylogenetic analyses were performed on two datasets of oribatid mite sequences. The first dataset comprised 26 sequences, which were aligned using Clustal with default parameters in the software (gap opening = 15, gap extension = 6.66). These parameters were found to yield the best-supported phylogeny. The second dataset consisted of 81 sequences, which were aligned using Clustal according to the parameter settings recommended in a previous study (gap opening = 20, gap extension = 0.1). The resulting alignment was manually trimmed to retain only homologous regions (522 bp). The best evolutionary models for phylogenetic analyses were selected using ModelFinder under the corrected Akaike Information Criterion (BICc). Phylogenetic tree inference was performed using the selected model, and nodal support was evaluated by bootstrap analysis with 1000 replicates [28]. The resulting phylogenetic trees were visualized and edited using the Interactive Tree of Life (iTOL) v.6. We initially performed multiple sequence alignment using MAFFT with default settings within the PhyloSuite software v1.2.3 platform. To further explore the impact of alignment on tree topology, we also generated a Bayesian phylogenetic tree based on the same MAFFT alignment using PhyloSuite. Detailed information on the sequences used in this study, including species names, GenBank accession numbers, and taxon, is provided in Supplementary Tables S1 and S2.

### 2.3. Transcriptome Sequencing and Assembly

#### 2.3.1. RNA Extraction

Fifty adult and nymphal *O.* cf. *terebrantis* were collected for de novo transcriptome sequencing. To eliminate potential interference of gut contents on transcriptomic sequencing results, the *O. terebrantis* were starved at room temperature for 24 h, then rapidly frozen in liquid nitrogen and stored at −80 °C for subsequent experiments. Total RNA was extracted using the Total RNA Extractor (Trizol) kit (B511311, Sangon Biotech Co., Ltd., Shanghai, China) according to the manufacturer’s protocol and treated with RNase-free DNase I to remove genomic DNA contamination. RNA integrity was evaluated with a 1.0% agarose gel. Thereafter, the quality and quantity of RNA were assessed using a NanoPhotometer^®^ spectrophotometer (IMPLEN, Westlake Village, CA, USA) and a Qubit^®^2.0 Flurometer (Invitrogen, Carlsbad, CA, USA). High-quality RNA samples were subsequently submitted to Sangon Biotech (Shanghai, China) for library preparation and sequencing.

#### 2.3.2. Library Preparation and Sequencing

A total amount of 1 μg of RNA per sample was used as input material for the RNA sample preparation. Sequencing libraries were generated using VAHTSTM mRNA-seq V2 Library Prep Kit for Illumina^®^ following the manufacturer’s recommendations, and index codes were added to attribute sequences to each sample. Briefly, mRNA was purified from total RNA using poly-T oligo-attached magnetic beads. Fragmentation was carried out using divalent cations under elevated temperature in VAHTSTM First Strand Synthesis Reaction Buffer (5X). First-strand cDNA was synthesized using random hexamer primer and M-MuLV Reverse Transcriptase (RNase H−). Second-strand cDNA synthesis was subsequently performed using DNA polymerase I and RNase H. Remaining overhangs were converted into blunt ends via exonuclease/polymerase activities. After adenylation of the 3′ ends of DNA fragments, an adaptor was ligated to prepare for library. In order to select cDNA fragments of preferentially 150–200 bp in length, the library fragments were purified with the AMPure XP system (Beckman Coulter, Beverly, MA, USA). Then 3 μL USER Enzyme (NEB, Ipswich, MA, USA) was used with size-selected, adaptor-ligated cDNA at 37 °C for 15 min, followed by 5 min at 95 °C before PCR. Then, PCR was performed with Phusion High-Fidelity DNA polymerase, Universal PCR primers, and Index (X) Primer. At last, PCR products were purified (AMPure XP system), and library quality was assessed on the Agilent Bioanalyzer 2100 system. The libraries were then quantified and pooled. Paired-end sequencing of the library was performed on the NovaSeq sequencers (Illumina, San Diego, CA, USA).

#### 2.3.3. Data Processing

FastQC (v0.11.2) was used to evaluate the quality of the sequencing data. Raw sequencing data were filtered using Trimmomatic (v0.36) to obtain clean data for downstream analyses [29]. The clean data were then assembled de novo into transcripts using Trinity (v2.4.0) with the parameter min_kmer_cov set to 2 and other parameters set to default [30]. The completeness of the assembly was assessed based on single-copy orthologs using BUSCO (v5.3.2). Redundancy among the transcripts generated by Trinity (v2.4.0) was reduced by selecting the longest transcript from each transcript cluster as a Unigene, which served as the reference sequence for subsequent analyses. Homology searches were performed against the CDD, KOG, NR, NT, PFAM, and GO databases using NCBI Blast v2.6 [31]. Annotation of orthologous groups in the Kyoto Encyclopedia of Genes and Genomes (KEGG) was carried out using the KAAS v2.1 tool [32,33].

## 3. Results

### 3.1. Habitat Investigation

**Figure 1:**
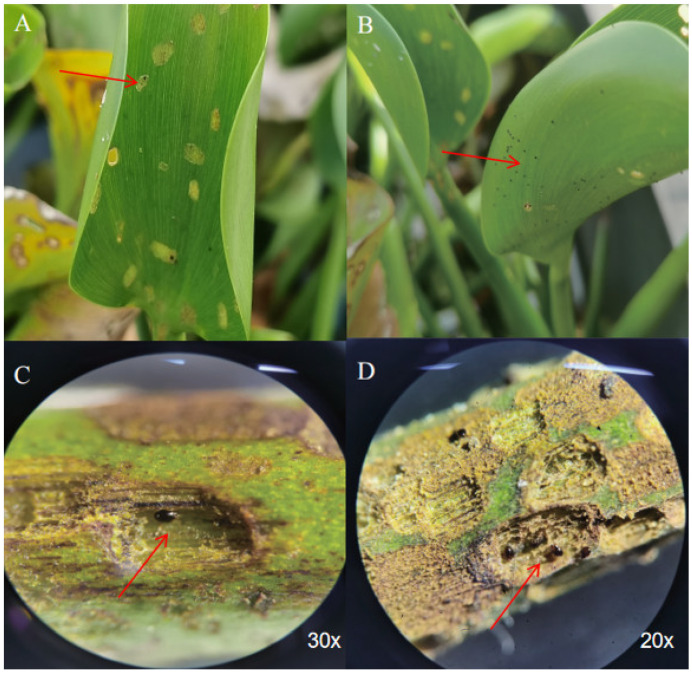
Ecological Distribution and Microscopic Morphology of *O.* cf. *terebrantis*. (**A**,**B**) The distribution of *O.* cf. *terebrantis* on water hyacinth leaves; (**C**,**D**) Morphological Characteristics of *O.* cf. *terebrantis* in water hyacinth Stems Observed under Stereo Microscopy. Note: (**C**): Optical magnification: 30× (objective lens: 3×; eyepiece lens: 10×); (**D**): Optical magnification: 20× (objective lens: 2×; eyepiece lens: 10×).

The *O.* cf. *terebrantis* inhabits the aquatic plant water hyacinth. Samples were collected at Minjiang University, Fujian Province, China, between 2024 and 2025. The habitat is characterized by a warm, humid subtropical monsoon climate, with an annual mean temperature of 19.6 °C and an annual precipitation of 1342.5 mm. Photographs of its habitat and images taken under a microscope of its morphology are provided. Observations under a stereomicroscope revealed that the oribatid mites fed on the mesophyll and stem tissues of water hyacinth, leaving irregular feeding cavities (Figure 1A,B). The morphological characteristics are as follows: the body color is glossy black; the idiosoma is divided into an anterior prosoma and a posterior opisthosoma; a pair of clavate sensilla is located laterally on the prosoma; and four pairs of legs are present (Figure 1C,D).

### 3.2. Scanning Electron Micrographs of the Microscopic Structure

**Figure 2:**
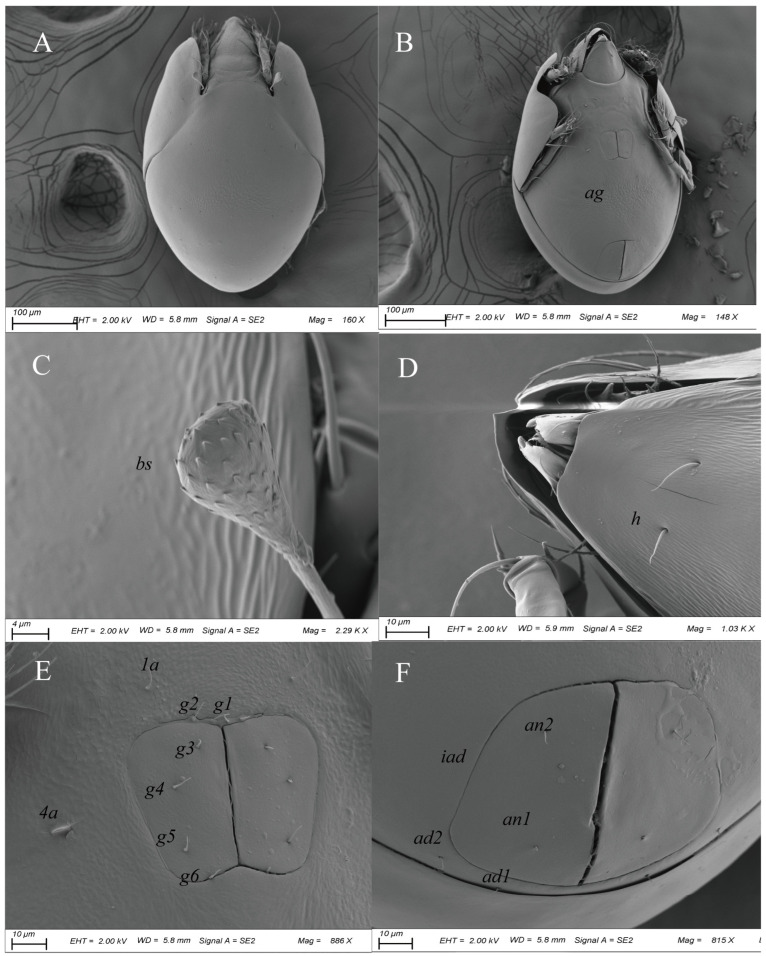
SEM micrographs of *O.* cf. *terebrantis*. adult. (**A**) dorsal view; (**B**) ventral view; (**C**) bothridial seta; (**D**) subcapitulum; (**E**) genital plate; (**F**) anal plate.

We compared the adult stage of the mites collected in this study with the original description of *Orthogalumna terebrantis* by Wallwork [34]. The micro-morphological features observed under SEM corresponded well with the published characteristics; however, pending complete life cycle data, we tentatively identify it as *O* cf. *terebrantis*. The detailed morphological features are described as follows. Body size 430 ± 20 μm in length and 270 ± 20 μm in width. Body color is glossy black. Body surface, pteromorphs, and subcapitular mentum with dense microgranules. Rostrum narrowly rounded. The prodorsum has a pair of bothridial seta (bs) (Figure 2A,B). A pair of movable, ear-shaped pteromorphs is present. Bothridial seta (bs)with a long stalk and a fusiform head bearing sparse minute spinules (Figure 2C). Chelicera chelate-dentate, with two setiform cheliceral setae inserted laterally on the segment (Figure 2D). The idiosoma is divided into the prodorsum and the notogaster by the sejugal suture, with the prodorsum and notogaster being non-articulating. The genital plate has six pairs of genital (ag1, ag2, ag3, ag4, ag5, ag6) (Figure 2E), one pair of aggenital (ag). Anal plates have two pairs of analand (an1, an2), a pair of iad, three pairs of adanal setae (ad1, ad2, ad3), setiform, thin, and smooth (Figure 2F).

### 3.3. Phylogenetic Analysis Based on 18S rDNA

The 18S rDNA sequence of *O.* cf. *terebrantis* was obtained from specimens sampled in Fujian Province, China. The sequence is 541 bp in length with a GC content of 45%. 6%. Phylogenetic analysis of *O.* cf. *terebrantis* was conducted using 18S rDNA sequences. The best-fit nucleotide substitution model for the ML tree was K2 + G as the optimal model for the dataset. The final alignment included 81 taxa, all of which are placed within the suborder Oribatida. This suborder is currently divided into six supercohorts: Palaeosomatides, Enarthronotides, Parhyposomatides, Mixonomatides, Nothrina, and Brachypylina.

**Figure 3:**
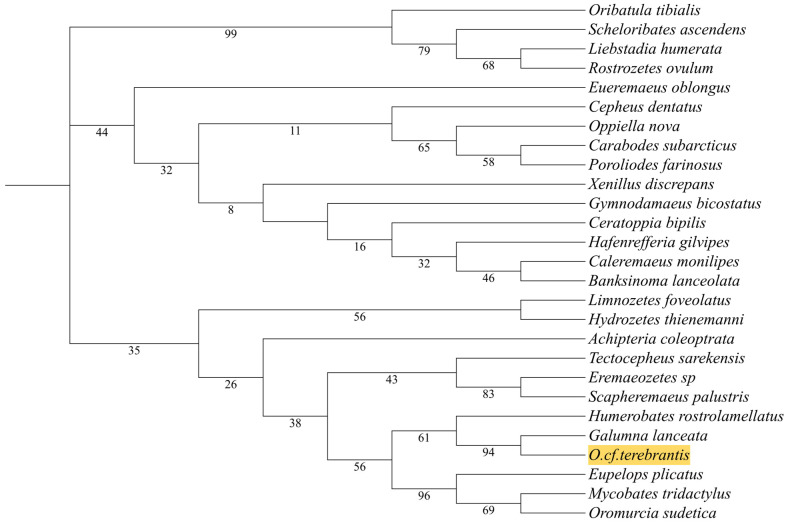
Maximum-likelihood tree based on 18S rDNA gene of Oribatida. Phylogenetic tree constructed from 18S rDNA sequences, showing relationships between *O.* cf. *terebrantis* and related taxa. Branch labels indicate support values (1000 replicates). In the phylogenetic tree, the *O.* cf. *terebrantis* in this study are highlighted with a yellow background.

In the ML tree, most taxa formed distinct, well-supported terminal branches, which resolved the phylogenetic position of *O.* cf. *terebrantis*. The 26 taxa are supercohort Brachypylina. The 18S rDNA phylogeny supports the placement of the Chinese specimens within Galumnoidea, but the short fragment length and low support at several deeper nodes limit the resolution of interspecific relationships (Figure 3); the *O.* cf. *terebrantis* was placed within Galumnoidea and formed a well-supported sister-group relationship with *Galumna lanceata* (bootstrap support = 94%). The ML tree (80 sequences + target, Clustal alignment) is now provided as Figure S1. Additionally, a Bayesian tree based on 81 sequences, aligned with MAFFT, is presented in Figure S2. The Bayesian tree showed an overall topology consistent with the maximum likelihood tree. The *O.* cf. *terebrantis* clustered with *Galumna lanceata*, and this clade was placed within the family Galumnidae.

### 3.4. De Novo Transcriptome Sequencing and Assembly

#### 3.4.1. 1Illumina Sequencing and De Novo Assembly

**Figure 4:**
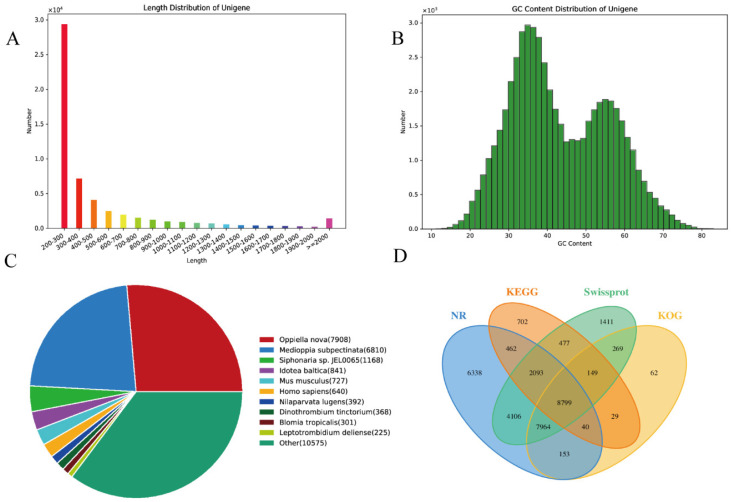
Summary statistics of the *O.* cf. *terebrantis* transcriptome assembly and annotation. (**A**) Length distribution of assembled unigenes; (**B**) GC content distribution of all unigenes; (**C**) Species distribution of top BLAST hits against the Nr database; (**D**) Functional annotation results across major public databases.

Total RNA was extracted from *O.* cf. *terebrantis* and utilized for library construction. Sequencing was performed on an Illumina NovaSeq platform (San Diego, CA, USA), yielding 6.7 Gb of raw data consisting of 45,195,624 paired-end reads. After rigorous quality control to filter adapter-contaminated and low-quality sequences, 40,827,552 clean reads (6.06 Gb) remained for analysis. De novo assembly via Trinity software v2.4 resulted in 55,110 unigenes, exhibiting an average length of 500.46 bp, an N50 of 686 bp, and a GC content of 41.99% (Figure 4A,B). The GC content of filtered transcripts clearly exhibited a bimodal distribution. The likely taxonomy of the origin of these transcripts was obtained by database searches. BUSCO analysis revealed a high level of completeness, with 74.5% of the conserved orthologs being present. The first high-quality transcriptome resource for *O.* cf. *terebrantis* was generated in this study (Table 1).

#### 3.4.2. Functional Annotation and Classification of Unigenes

A total of 55,110 annotated unigenes of *O.* cf. *terebrantis* were compared to known species using the NCBI Non-Redundant (NR) database. In terms of taxonomic distribution, the sequences exhibited the highest similarity to *Oppiella nova*, followed by *Medioppia subpectinata* (Figure 4C). Furthermore, the assembled unigenes were annotated against seven major functional databases, including Nr, Nt, GO, COG, KEGG, SwissProt, and InterPro (encompassing CDD and PFAM). Overall, 36,589 unigenes (66.39% of the total) were successfully annotated. Specifically, the numbers of unigenes annotated in each database were as follows: 29,955 (54.35%) in Nr, 11,767 (21.35%) in Nt, 25,268 (45.85%) in SwissProt, 12,751 (23.14%) in KEGG, 17,465 (31.69%) in KOG, 12,311 (22.34%) in CDD, 13,682 (24.83%) in PFAM, and 21,267 (39.24%) in GO (Table 1).

**Figure 5:**
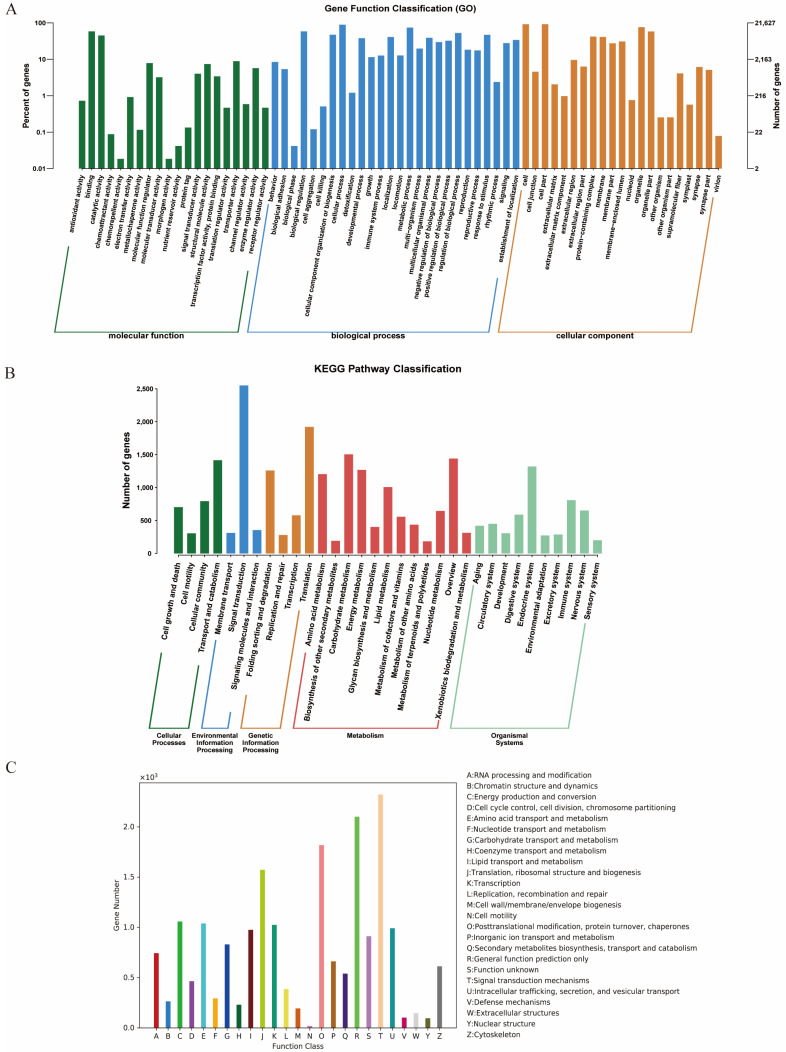
Functional classification of unigenes in *O.* cf. *terebrantis*. (**A**) GO functional classification of unigune; (**B**) KEGG pathway classification of unigune; (**C**) KOG functional class of unigune.

The GO analysis identified 21,627 transcripts linked to at least one GO term. The main categories with more individually annotated transcripts were cellular component, biological process, and molecular function (Figure 5A). Additionally, 12,751 proteins were assigned to various KEGG pathways. KEGG pathway annotation revealed that the mapped genes were mainly involved in environmental information processing, genetic information processing, metabolism, and organismal systems (Figure 5B). Among these categories, the signal transduction pathway contained the largest number of genes. This was followed by pathways related to translation, carbohydrate metabolism, the overview category, and environmental adaptation. The results indicate that genes in this sample are largely engaged in signal transmission, protein synthesis, substance metabolism, and responses to the environment. A total of 17,465 predicted proteins were annotated by the KOG database and assigned to 25 functional categories of KOG. Based on the KOG annotation results presented in the figure, the functional landscape of the annotated genes reveals a primary investment in core cellular maintenance and regulatory processes. (Figure 5C). Functional annotation of the transcriptome revealed a high enrichment of genes involved in core metabolic and signal transduction processes, providing a foundational dataset for ecological studies of host interactions and for gene discovery in this species. The complete annotation results, including GO term assignments, KEGG pathway mappings, and KOG functional categories for all sequences, are provided in Supplementary Table S3.

## 4. Discussion

In this study, we provide the first integrative characterization of *O.* cf. *terebrantis* in China. These results establish a foundational resource for oribatid mites and provide a molecular entry point to dissect the mechanisms underlying their host association with water hyacinth.

Published literature on Galumnidae has predominantly focused on morphological descriptions of new species and taxonomic revisions [35,36,37,38,39,40]. While these foundational studies have substantially advanced our understanding of oribatid mite diversity, purely morphology-based approaches exhibit inherent limitations in detecting cryptic species and resolving complex phylogenetic relationships. Consequently, interdisciplinary methodologies have become essential for accurate species delimitation and evolutionary inference [6]. The application of scanning electron microscopy (SEM) has markedly enhanced taxonomic precision by revealing fine-scale cuticular ultrastructure previously inaccessible with light microscopy [41,42].

With the advent of molecular techniques, mitochondrial cytochrome c oxidase subunit I (COI) and nuclear ribosomal RNA genes (18S, 5.8S, 28S), together with the internal transcribed spacer (ITS) regions, have been increasingly employed for molecular identification and phylogenetic reconstruction in acarological research [43,44]. In the present study, SEM micrographs provide high-resolution morphological documentation of the newly recorded Chinese population of *O.* cf. *terebrantis*, with these ultrastructural features serving as critical diagnostic characters for taxonomic placement within Galumnidae. Phylogenetic analysis based on 18S rDNA robustly positions *O.* cf. *terebrantis* within the family and establishes a well-supported sister-group relationship with *Galumna lanceata*. This finding is consistent with and extends the work of Pachl [26], contributing a pivotal data point to the emerging molecular phylogenetic framework for this species-rich genus.

The ecological observations that *O. terebrantis* forms feeding cavities within mesophyll and stem tissues of water hyacinth, coupled with its high local abundance, align with earlier reports that this mite can substantially reduce host photosynthetic performance and growth [8]. In the context of classical biological control programs that deploy *O. terebrantis* alongside specialist weevils such as *Neochetina eichhorniae* and *N. bruchi*, our data support the view that the mite may act as an additional stressor that exacerbates host decline when integrated into multi-agent strategies [9]. Importantly, documenting its presence in Chinese waterways indicates that the species has already established beyond previously documented South American and other introduced ranges, raising the need to evaluate both its control potential and any unintended ecological consequences in Asian invaded systems [10,11,12]. The transcriptome resource offers several lines of mechanistic insights into the biology of *O. terebrantis* as a specialist herbivore of aquatic macrophytes. The high proportion of genes annotated in GO, KEGG, and KOG categories related to signal transduction, environmental adaptation, and core metabolic processes is consistent with transcriptomic profiles reported in other acarine species that engage in complex host interactions and niche specialization [16,23,25]. Enrichment of pathways linked to signal transduction and translation suggests substantial investment in sensory processing and rapid protein turnover, which may underpin the ability of *O.* cf. *terebrantis* to locate suitable tissues within aquatic hosts and respond dynamically to fluctuating environmental conditions in lotic habitats. Comparison with transcriptomic studies of other mites underscores both shared and distinctive features of *O.* cf. *terebrantis*. In poultry ectoparasites such as *Dermanyssus gallinae* and *Ornithonyssus sylviarum*, expansions of chemosensory receptor gene families have been linked to host-seeking behavior and blood-feeding adaptation [16,17]. By contrast, plant-associated mites such as *Tetranychus urticae*, *Eotetranychus sexmaculatus*, and *Amblyseius swirskii* exhibit pronounced enrichment of detoxification enzymes, plant cell wall-degrading enzymes, and effectors involved in plant defense modulation [18,23,24,25]. Although our annotation was performed at a global functional level, the observed dominance of environmental information processing and metabolic pathways in *O.* cf. *terebrantis* suggests convergence with other plant-associated mites, while also reflecting unique aspects of adaptation to submerged or semi-submerged plant tissues that differ from those of terrestrial hosts.

The oribatid mite transcriptome exhibited a bimodal GC distribution during quality control. Taxonomic annotation of the assembled contigs revealed no detectable signal from the host plant *E. crassipes*, despite 24 h of starvation being insufficient for complete gut clearance. Instead, only trace reads (1–3 per species) mapped to model plants (*Arabidopsis thaliana*, *Oryza sativa*, *Nicotiana tabacum*, *Solanum lycopersicum*), suggesting trace laboratory cross-contamination rather than residual host tissue. We attribute the absence of water hyacinth transcripts to two factors: the chromosome-level *E. crassipes* genome was deposited in NCBI only in 2024 [45], and our annotation database likely lacked this species; and the poly(A) –enrichment library preparation poorly captures degraded plant mRNA with shortened poly-A tails from the mite gut, further reducing detection probability. Bimodal GC distributions are common in mixed-origin transcriptomes. Videvall [46] resolved GC bimodality in Plasmodium ashfordi into superimposed host (high-GC) and parasite (low-GC) transcript pools, while Liu and Sun [47,48] reported analogous patterns in radiolarian and ciliate holobionts arising from GC divergence among coexisting organisms. Oribatid mites harbor diverse symbionts (algae, bacteria, fungi) whose genomic GC contents (30–70%) may differ substantially from the arthropod host (~32–42%), providing a biological basis for the observed pattern. The technical artifacts must also be considered. rRNA-depletion inefficiency, batch-specific reagent variation, and PCR amplification bias can introduce GC deviation, with residual rRNA forming a secondary peak due to its distinct GC composition [49,50]. Given these uncertainties, we recommend taxonomic filtering (retaining only Arthropoda-annotated contigs) for downstream analyses to exclude potential contaminants and symbiont-derived sequences.

Beyond its taxonomic and evolutionary implications, our work has direct relevance for the management of water hyacinth, one of the most damaging aquatic invasive plants worldwide. Biological control programs increasingly favor multi-agent assemblages that combine herbivorous insects and mites to achieve sustained suppression of water hyacinth biomass while minimizing chemical inputs. By providing a genomic framework for *O.* cf. *terebrantis*, our study lays the groundwork for identifying molecular markers of population structure, host specificity, and potential non-target risks, which are essential parameters for risk assessment and for optimizing release strategies in different regions.

Before concluding, we acknowledge several limitations that may affect the interpretation of our findings. First, phylogenetic inference is based on a single nuclear marker (18S rDNA), which, while informative, may not fully resolve relationships among closely related species. Future studies should leverage the extensive transcriptome data generated here to design multi-gene panels or conduct phylogenomic analyses for a more definitive resolution. Second, our functional interpretations from transcriptomic data are based on homology and pathway enrichment; they are predictive and require validation through experimental approaches.

## 5. Conclusions

In conclusion, this study presents the first comprehensive dataset integrating ecological, morphological, phylogenetic, and transcriptome resources for *O.* cf. *terebrantis*. The high-quality transcriptome assembly fills a critical gap in genomic resources for Acari, while the SEM observations and habitat data provide essential context for interpreting its biology. The phylogenetic framework will facilitate comparative studies within this lineage. Together, these data elucidate the molecular basis of host adaptation and underscore the power of integrative approaches in studying non-model organisms. Transcriptome resource of *O.* cf. *terebrantis* will serve as a foundation for subsequent functional genomics studies and may inform biocontrol efforts targeting invasive aquatic plants.

## Figures and Tables

**Table 1:** Summary statistic of the Transcriptome sequencing and sequence assembly of *O.* cf. *terebrantis*.

Assembly Statistics	Transcriptome
Assemble size (bp)	27,580,367
Sequencing average length (bp)	500.46
N50 (bp)	686
Q20 (%)	98.50
GC content (%)	41.99
Annotated in CDD	12,311
Annotated in PFAM	13,682
Annotated in KEGG	12,751
Annotated in KOG	17,465
Annotated in SwissProt	25,268
Annotated in GO	21,627
Annotated in NR	29,955
Annotated in NT	11,767
Annotated in at least one database	36,589
Annotated in all databases	1506
Total genes	55,110
BUSCO	74.5%
Complete	190 (74.5%)
Complete and single copy	175 (68.6%)
Duplicated	15 (5.9%)
Fragmented	42 (16.5%)
Missing	23 (9.0%)
Total	255

## Data Availability

The 18SrDNA gene sequence and raw transcriptome data generated in this study are available in the NCBI databases. Specifically, the 18SrDNA sequence is accessible via GenBank (accessionPX898667), and the raw reads are available in the Sequence Read Archive (SRA). BioProject, BioSample, and SRA ID are PRJNA1406171, SAMN54763567, and SRR36932054.

## Supplementary Materials

The following supporting information can be downloaded at: https://www.mdpi.com/article/10.3390/cimb48060619/s1.
