# Supplementary figures and images for: Identification and Transcriptome Resource of the Mite Orthogalumna cf. terebrantis (Acari: Galumnidae) in China
Source: Curr Issues Mol Biol. 2026 Jun 15;48(6):619. doi: 10.3390/cimb48060619 (PMC13298155; doi:10.3390/cimb48060619)

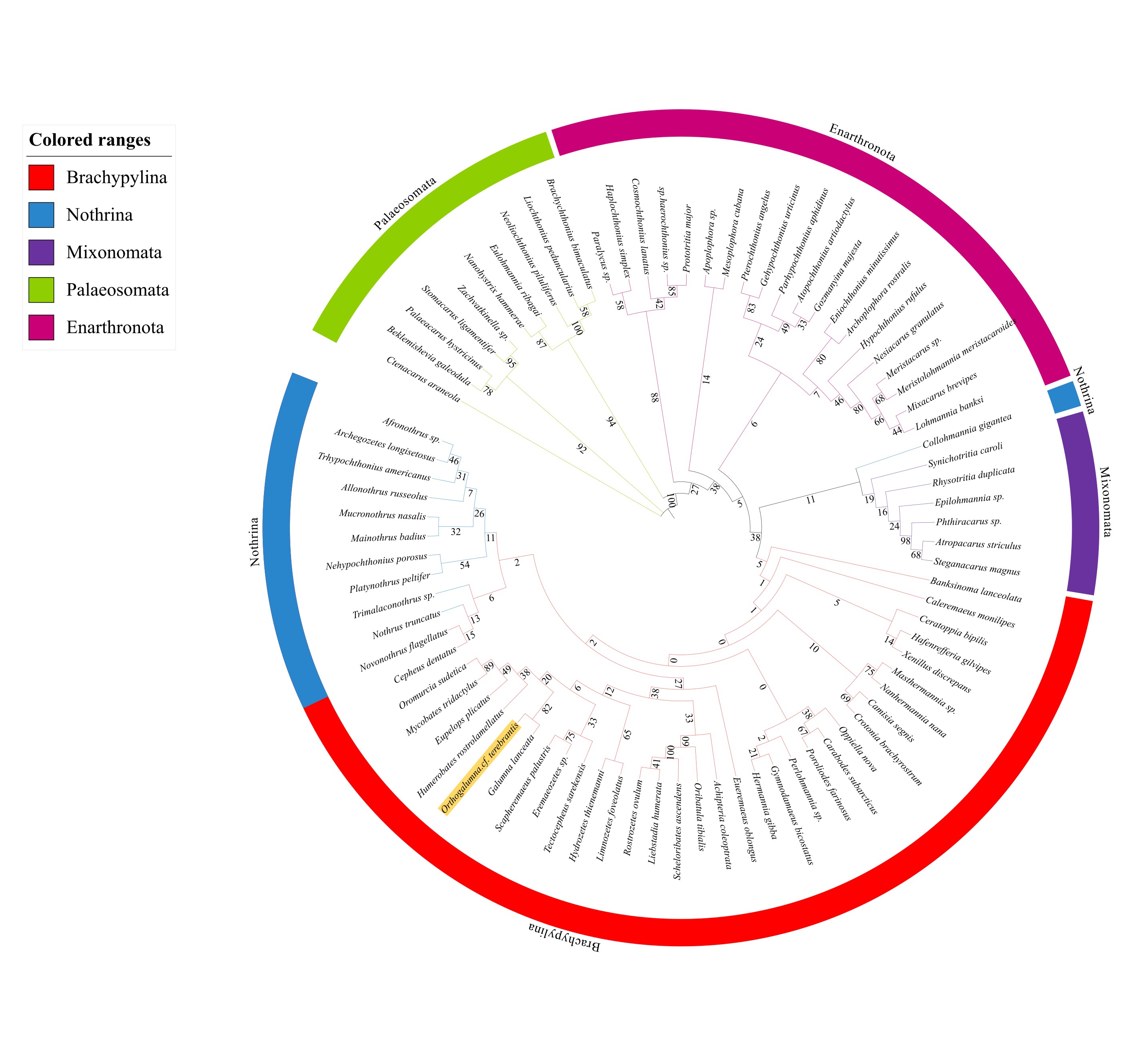

Supplement: Supplementary file 1 [file cimb-48-00619-s001.zip › Fig S1 Maximum Likelihood (ML) phylogenetic tree inferred from 81 nuclear 18S rDNA sequences of oribatid mites (Oribatida).jpeg]

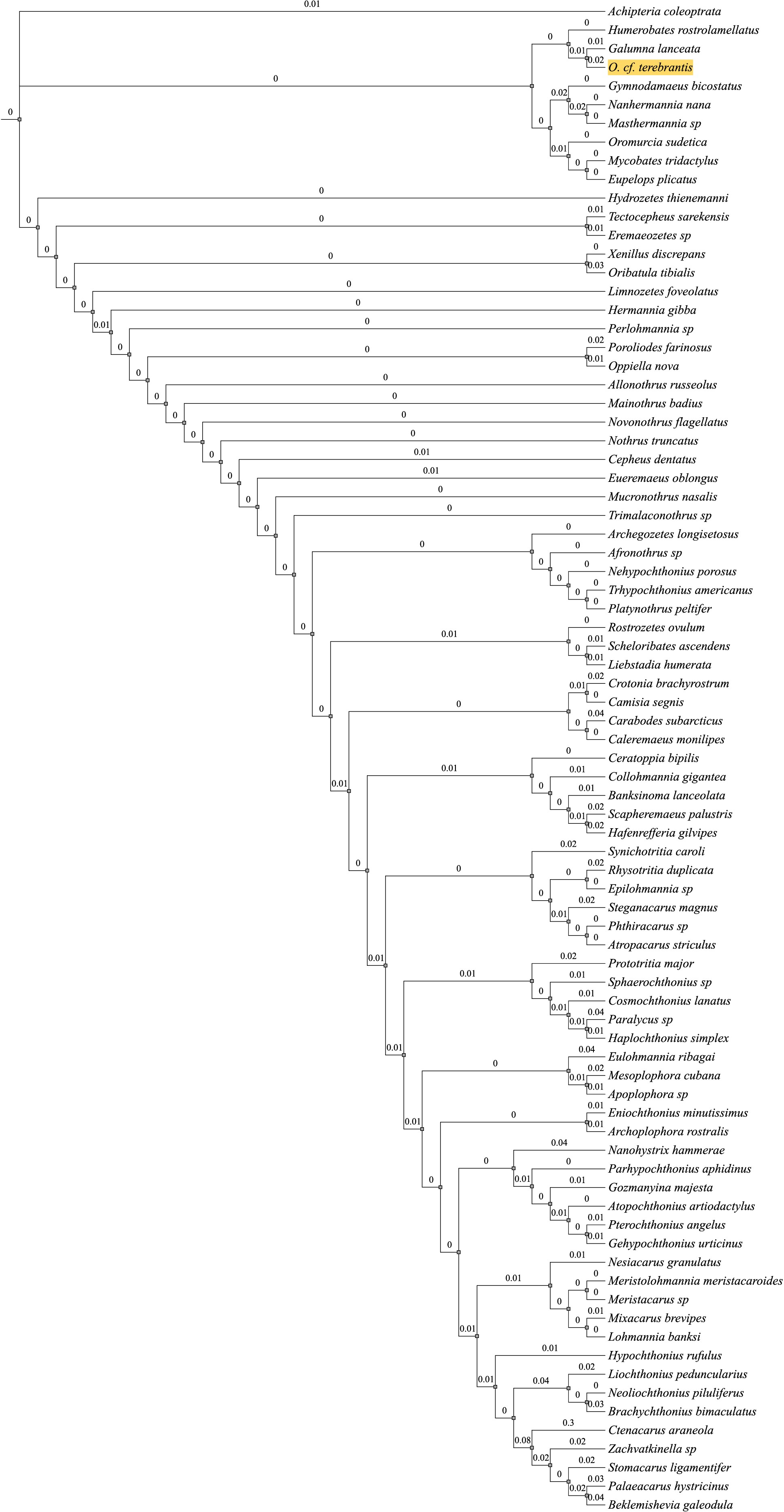

Supplement: Supplementary file 1 [file cimb-48-00619-s001.zip › Fig S2 Bayesian phylogenetic tree inferred from 81 nuclear 18S rDNA sequences of oribatid mites (Oribatida).jpeg]
